# Microtubule motor driven interactions of lipid droplets: Specificities and opportunities

**DOI:** 10.3389/fcell.2022.893375

**Published:** 2022-09-19

**Authors:** Jagjeet Singh, Paulomi Sanghavi, Roop Mallik

**Affiliations:** ^1^ Department of Biological Sciences, Tata Institute of Fundamental Research, Mumbai, India; ^2^ Department of Biosciences and Bioengineering, Indian Institute of Technology Bombay, Mumbai, India

**Keywords:** microtubule motor, kinesin, dynein, lipid droplet, membrane contacts, lipid metabolism, pathogen, drug delivery

## Abstract

Lipid Droplets (LDs) are evolutionarily conserved cellular organelles that store neutral lipids such as triacylglycerol and cholesterol-esters. Neutral lipids are enclosed within the limiting membrane of the LD, which is a monolayer of phospholipids and is therefore fundamentally different from the bilayer membrane enclosing most other organelles. LDs have long been viewed as a storehouse of lipids needed on demand for generating energy and membranes inside cells. Outside this classical view, we are now realizing that LDs have significant roles in protein sequestration, supply of signalling lipids, viral replication, lipoprotein production and many other functions of important physiological consequence. To execute such functions, LDs must often exchange lipids and proteins with other organelles (e.g., the ER, lysosomes, mitochondria) *via* physical contacts. But before such exchanges can occur, how does a micron-sized LD with limited ability to diffuse around find its cognate organelle? There is growing evidence that motor protein driven motion of LDs along microtubules may facilitate such LD-organelle interactions. We will summarize some aspects of LD motion leading to LD-organelle contacts, how these change with metabolic state and pathogen infections, and also ask how these pathways could perhaps be targeted selectively in the context of disease and drug delivery. Such a possibility arises because the binding of motor proteins to the monolayer membrane on LDs could be different from motor binding to the membrane on other cellular organelles.

## Introduction

Lipids are an integral component of living systems. In addition to their roles of storing energy and forming cell membranes, lipids have crucial roles in cell signaling (Hannun and Obeid, 2008). Almost all types of cells have the capability to accumulate lipids, with the mechanism of lipid storage being fairly conserved from yeast to mammals and plants (Zhang and Liu, 2017). Lipid Droplets (LDs) are the cellular organelles that specialize in storing and supplying lipids. LDs have a unique structure consisting of a hydrophobic core, containing predominantly triacylglycerol (TG) and steryl esters, coated by a phospholipid monolayer which emulsifies the LD in the cytoplasm. LD-specific proteins, including motor proteins, are recruited to this monolayer from the cytosol or from other membranous organelles (e.g., the endoplasmic reticulum; ER) by mechanisms that are under intense investigation (Olarte et al., 2022). We are now beginning to appreciate how LDs interact with other cellular compartments, and how these interactions play a role in lipid metabolism (Renne and Hariri, 2021). The membrane phospholipids of the LD, along with their set of bound proteins, control storage and consumption of the LD contents for generating energy, membrane and signalling lipids (Walther and Farese, 2012). Dysfunctions in the pathways that control LD biogenesis and/or catabolism can therefore have severe physiological consequences (Greenberg et al., 2011).

Almost all kinds of cells contain LDs, but the mechanisms that allow the cell to access to these lipids can be different across cell types. Adipocytes are the archetypal cells for LD storage. Most of the cell volume in white adipocytes is occupied by a few large LDs, leaving little space for these LDs to move around. The major lipases that are known to catabolize LDs in adipocytes, namely Adipose triglyceride lipase-ATGL and Hormone sensitive lipase-HSL, are located in the cytoplasm of these cells (Miyoshi et al., 2008). A co-activator of ATGL (Comparative gene identification-58; CGI-58), which is also cytosolic, is required to recruit the cytosolic lipase to LDs for lipolysis to proceed. Therefore, the mechanisms that activate LD-lipolysis may not be highly sensitive to the intra-cellular localization of LDs in these cells. In contrast, hepatocytes in the liver have much smaller LDs (1–2 micron diameter). Interestingly, the major lipase (Carboxylesterase 3/triacylglycerol hydrolase; Ces3/TGH) that channels LD-contents towards producing lipoproteins in the liver is not cytosolic. Rather, Ces3/TGH is found on the endoplasmic reticulum (ER) of hepatocytes (Lehner et al., 2012). In hepatocytes, therefore, active motion may be required to localize the micron-sized LDs to the ER, so that LD-ER contacts can form for Ces3/TGH to access the LDs (Rai et al., 2017). Similar to hepatocytes, most cell-types have LDs that are smaller than adipocytes. Nevertheless, diffusion of LDs is still highly restricted in these cells. Though this aspect remains to be explored broadly, it is conceivable that LD-organelle interactions would require motor protein driven “delivery” of LDs to diverse intracellular locations in these cell-types.

The motor-protein driven motion of LDs has been discussed in excellent reviews (Welte, 2015; Welte and Gould, 2017; Kilwein and Welte, 2019), and will be summarized briefly here. Directed LD motion has been reported in hepatocytes, fibroblasts, kidney epithelial cells, adrenal cortex cells etc. (Targett-Adams et al., 2003; Nan et al., 2006; Turró et al., 2006; Barak et al., 2013). Motion of LDs in *Drosophila* embryos has been studied extensively and is known to change across developmental stages (Welte, 2015). The microtubule minus end-directed motor cytoplasmic dynein is present on LDs (Gross et al., 2000). Dynein inactivation and/or microtubule depolymerization blocks the formation of LD-complexes in NIH3T3 cells, raising the possibility of growth/fusion of LDs facilitated by dynein (Boström et al., 2005). The plus-end directed microtubule motor Kif5b (Kinesin-1) is associated with LDs in hepatocytes and *Drosophila* embryos (Turró et al., 2006; Shubeita et al., 2008; Rai et al., 2017). Another study showed that kinesin and dynein work together to mediate LD motion in A549 cells (Sims and Xie, 2009). In some cases, myosin family motors have also been reported to drive LD motility. Myo2p, a class V myosin has been known to drive LDs from mother to daughter cell in *Saccharomyces cerevisiae* (Knoblach and Rachubinski, 2015). A non-muscle myosin II can also move LDs indirectly by sliding actin filaments with LDs attached on them in zebrafish embryos (Dutta and Kumar Sinha, 2015).

Recent advances in imaging using label-free methods (Dou et al., 2012) or by using low-toxicity fluorescent dyes (Collot et al., 2018; Zheng et al., 2019) have allowed longer observation of LD dynamics with better spatial and temporal resolution. Multispectral imaging was used to quantify the interaction different cellular organelles in massively parallelized manner to conclude that LDs interact extensively with many other organelles, with LD-ER interactions being most promiscuous (Valm et al., 2017). The ubiquitous nature of LD motion and LD-organelle interactions in different cell types is clear from these studies. Keeping these advances in mind, there is now an urgent need to understand why LDs move in response to specific cellular/physiological stimuli, and how such stimuli activate the agents of LD motion (i.e., the motors). With better understanding of these mechanisms, it may also be possible to target LD motion selectively for therapeutic benefits (see later). We will discuss some aspects of microtubule motor driven LD motion relating to cellular physiology, health and disease with these motivations in mind.

### Lipid droplet–Organelle interactions: Regulation by motor proteins and physiological relevance

LDs can interact with other organelles through membrane contact sites, regions where inter-organellar communication can happen via lipid/protein exchange (Hugenroth and Bohnert, 2020; Renne and Hariri, 2021). Microtubule depolymerization changes the interactome of LDs drastically, possibly because it affects the co-localization of LDs with peroxisomes, mitochondria and lysosomes to abrogate lipid/protein exchanges (Valm et al., 2017). LDs provide fatty acids for β-oxidation in mitochondria to generate energy during nutrient deprivation. For lipid transfer to occur, LD-mitochondria interactions could be required, with this interaction requiring the Synaptosomal Associated Protein 23 or SNAP23 (Jägerström et al., 2009). Recent studies have shown that nutrient deprivation sensed by AMPK (AMP-activated kinase) reorganizes the network of detyrosinated microtubules in fibroblast cells. The kinesin-2 motor may drive LD motion upon starvation to bring LDs and mitochondria in close proximity, so that LDs can be consumed to generate fatty acids (Herms et al., 2015). We found that SNAP23 and a mitochondrial marker (VDAC-1) are significantly increased on LDs in hepatocytes within rat liver when the animal is fasted (Sadh et al., 2017). This supports the requirement of SNAP23 for LD-mitochondria interaction, suggesting a physiological angle to this finding.

**FIGURE 1:**
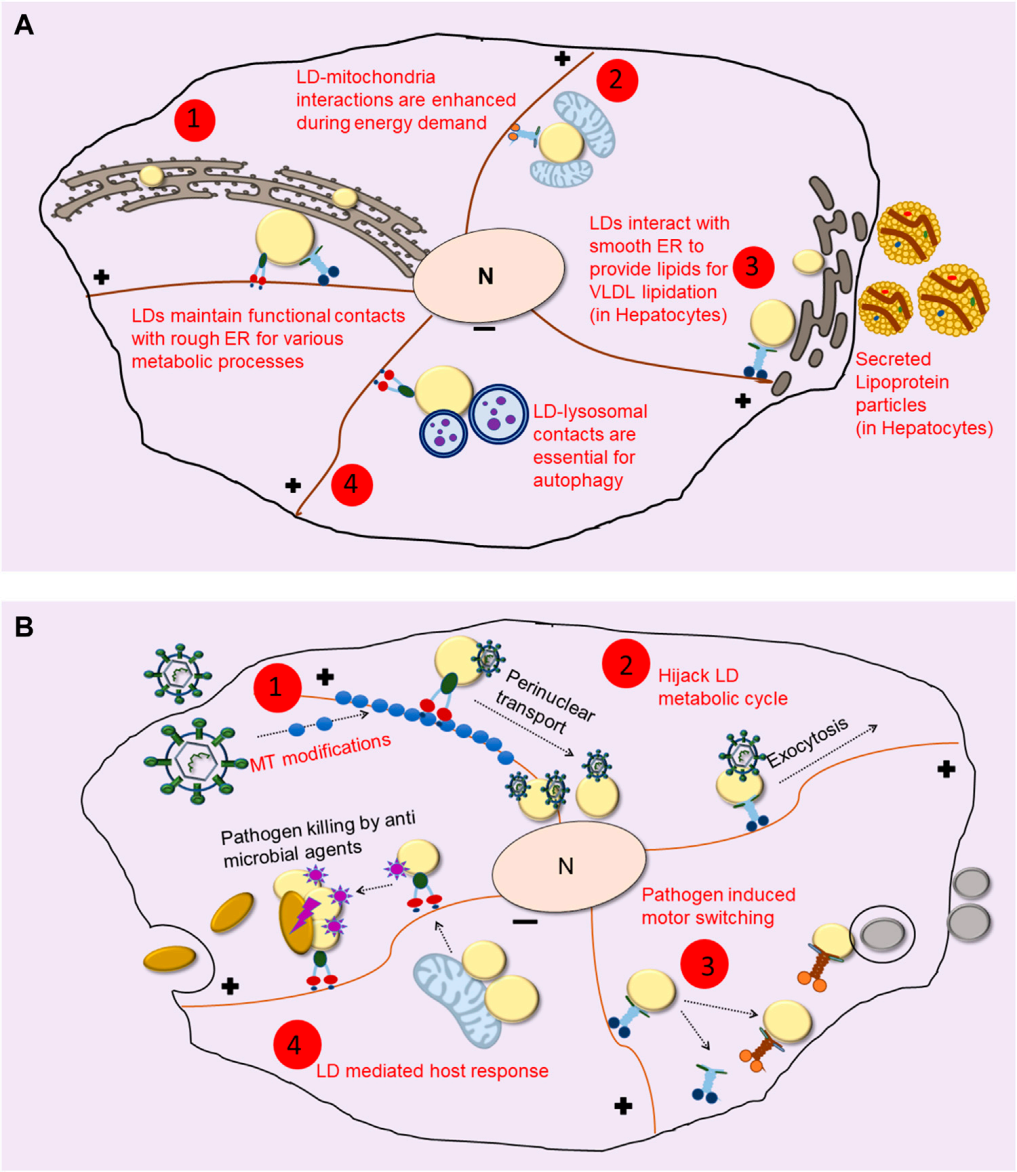
LDs Utilize the Microtubule cytoskeleton to Interact with other Organelles. **(A)** Even after its biogenesis from the ER, LDs maintain contacts with ER for protein and lipid trafficking during their life cycle (shown in 1). During nutrient deprivation and/or energy demand LDs interact with mitochondria for β-oxidation (as shown in 2). In hepatocytes, kinesin-1 drives LDs to smooth-ER at the cell periphery where it facilitates lipid transfer for lipidating VLDL particles which are then secreted out into blood for delivery to peripheral organs (depicted in 3). Extreme energy stress upregulates lipophagy, where LDs are engulfed by autophagosomes to derive energy and nutrients for cell survival (shown in 4). **(B)** Pathogens cause changes in LD dynamics and localization using different mechanisms. Some pathogens cause changes in microtubule (MT) organization and modifications (shown in 1) to cause altered motor driven LD transport. Viruses such as HCV hitchhike with LDs and hijack their metabolic cycle for their entry into the nucleus as well as for the exocytosis of viral particles (as shown in 2). LDs often show differential recruitment of motors upon infection (shown in 3) to cause LD localization to pathogen containing vacuole and other organelles. As opposed to these, a counter defense mechanism used by the host is accumulation of anti-microbial proteins on LDs, which then act as immune hubs and help in elimination of the pathogen (shown in 4).

The kinesin-1 motor drives LD transport towards the cell periphery in hepatocytes to facilitate interaction between LDs and the smooth-ER (sER) leading to the catabolism of LDs for providing TG towards very low density lipoprotein (VLDL) assembly in the sER (Rai et al., 2017; Kumar et al., 2019). Perhaps, therefore, two separate motor-dependent pathways exist for routing LD-contents to different organelles. First, a kinesin-2/SNAP23 dependent pathway in starved state that transports LDs to the mitochondria along detyrosinated microtubules, so as to derive energy by *β*-oxidation of LD contents in the mitochondria. Second, a Kif5b (kinesin-1) dependent pathway in nutrient-replete fed state where this motor transports LDs towards the peripheral ER in hepatocytes, where LD-TG is utilized for assembling VLDL particles as shown in Figure 1A (Rai et al., 2017; Kumar et al., 2019). Note that SNAP23 is also implicated in dynein mediated LD-LD fusion (Boström et al., 2007). Contrary to the conventional school of thought, a study on brown adipose tissue also demonstrated that mitochondria, instead of being the acceptor, can also provide fatty acid substrates to LDs for the synthesis of neutral lipids (Benador et al., 2018, 2019). The context-specific role of motors in mediating LD-mitochondria interactions is therefore open to further exploration.

Another system where LD motion has been linked to a specific physiological function is in mammary gland cells. Intravital imaging of a lactating mouse mammary gland revealed that LDs originate all over the cell and move towards the centrioles near the apical surface (Masedunskas et al., 2017). This motion is likely driven by dynein, a motor that was detected in a proteomic study of LDs from mouse mammary cells (Wu et al., 2000). LDs, in addition to being a lipid/energy reservoir, also act as a depot for protein trafficking. Quite remarkably, LDs sequester away histones from the nuclei of cells at early stages of development in the *Drosophila* embryo, and may then possibly deliver these histones for packaging DNA as development proceeds and cells multiply (Cermelli et al., 2006). More comprehensive discussions on LD-organelle interactions and their downstream consequences can be found (Thiam and Dugail, 2019; Herker et al., 2021), and will not be repeated here. As more sensitive techniques are developed to track these interactions and our understanding of LD motility expands, stronger connections between these fields may emerge. It is however clear at this stage that motor proteins bind to the LD membrane and directly drive LD motion in many situations.

### Recruitment of motor proteins to lipid droplets

How specific proteins are targeted to the LD is a topic of intense discussion (Olarte et al., 2022). Very interestingly, no dedicated machinery is known for this purpose. This is in contrast to other organelles, for example, the translocon machinery for the ER, or the phosphorylation of phosphatidylinositols that modulates protein composition in the endo-phagocytic pathway. It therefore appears likely that biophysical properties of the LD membrane (e.g., packing defects, phospholipids with different intrinsic curvatures etc.) control protein recruitment, and once recruited, a protein must compete to stay on the LD in the face of protein crowding. The so-called Class I proteins on LDs have hydrophobic segments that insert them on the ER membrane, from where they can get partitioned onto the LDs. This pathway is also called the ERTOLD (ER-to-LD) pathway for protein recruitment to LDs. Some examples of ERTOLD proteins are LD-associated hydrolase (LDAH), glycerol 3-phosphate acyltransferase 4 (GPAT4), Adipose TG lipase (ATGL) and Caveolin. Class II proteins, on the other hand, are cytosolic proteins with amphipathic helices that cause them to get recruited to LDs because the LD surface presents packing defects (CYTOLD; cytosol-to-LD pathway). Examples of CYTOLD proteins are perilipins, CTP:phosphocholine cytidylyltransferase (CCT) etc.

Some studies have also identified LD-resident proteins that interact with motors. A perilipin homologue LSD2 has been shown to coordinate directionality of LD transport in *Drosophila* embryos (Cohen, 2005; Welte et al., 2005). Another perilipin family protein PLIN3 coprecipitated with Dynein subunit Dync1i1 (dynein cytoplasmic 1 intermediate chain 1) and colocalized with the said subunit on the LDs (Gu et al., 2019). In *Drosophila* embryos absence of Klar alters the distribution of LDs inside the embryo and causes mis-localization of LDs to the yolk cell (Guo et al., 2005; Yu et al., 2011). Motors may also be recruited directly by the phospholipids present on the LD-monolayer without any adapter. We reported that kinesin-1 is recruited to LDs by direct binding to phosphatidic acid (PA) on the LD monolayer (Kumar et al., 2019). Our understanding of the motors that populate the LD membrane, how they change in response to cellular functions, and the pathway for their recruitment is still in its infancy.

### Motors in the liver: Systemic lipid homeostasis

The liver is one of the most metabolically active organs of the body in terms of lipid and fatty acid metabolism. The liver also plays an important role in maintaining the energetic status of other organs (Viollet et al., 2006). The parenchymal liver cells (hepatocytes) harbour a large number of LDs, making them a dynamic storehouse of triglycerides and steryl esters. These triglycerides can be repackaged into VLDL and secreted out in blood for use as an energy/lipid source in other organs. A majority of the lipids used for VLDL lipidation in hepatocytes are derived from cytosolic LDs (Wiggins and Gibbons, 1992). It was known for a long time that depolymerization of microtubules decreases VLDL lipidation in hepatocytes (Reaven and Reaven, 1980). We found that LDs purified from rat liver are transported robustly by kinesin-1 along microtubules when the LDs are prepared from a fed animal, but not from a fasted animal (Barak et al., 2013). Follow-up studies reported that this motion is brought about by binding of kinesin-1 to phosphatidic acid (PA) on the LD monolayer (Kumar et al., 2019). Most importantly, PA is generated on the LD membrane downstream of insulin signalling, thereby explaining the dependence of LD motion on Fed/Fasted state of the animal. Activation of phospholipase D1 (PLD1) by insulin signalling converts phosphatidylcholine to phosphatidic acid (PA) on the LD membrane (Kumar et al., 2019). Kinesin-1 then binds to PA on LDs, transporting LDs to the periphery of hepatocytes, where contacts form between LDs and the ER network. These contacts may allow LD-to-ER transfer of lipids to assemble VLDL in the ER lumen (Rai et al., 2017). Fasting causes TG breakdown in adipocytes and consequently increased free fatty acid (FFA) flux in blood circulation. The liver absorbs these potentially toxic FFAs from circulation and stores them away as triglycerides inside LDs in hepatocytes so as to protect other organs from lipotoxicity (Bradbury, 2006; Takahashi et al., 2010). Lowered insulin signaling in fasted state reduces the recruitment of kinesin-1 to LDs, thus lowering the triglyceride supply for VLDL lipidation in a homeostatic manner. This fine-tuning likely safeguards other organs from lipotoxic effects of fatty acids in fasted state (Rai et al., 2017), a possibility that was demonstrated later by us (Kumar et al., 2019).

Very recently, we reported an assay to quantify LD-ER interactions *in-vitro*, where an ER-mimicking proteinacous membrane was deposited on a coverslip in the form of a supported lipid bilayer, and LDs purified from liver were found to physically tether to this ER-mimic (Kamerkar et al., 2022). Quite remarkably, two molecular factors (the Rab18 GTPase and PA) promoted LD-ER tethering across the Fed/Fasted transition as well as when an immune response was activated in the animal. These results provide an assay to investigate LD-resident proteins (motors or non-motors) that can control physiological responses. Taken together, these findings may allow us to make headway in deciphering some of the mechanisms behind lipid disorders, metabolic syndrome and pathogen infections.

### Lipid droplet motion and disease

Although a clear mechanistic connection is lacking, abnormal motion of LDs correlates with genetic, metabolic and lifestyle-related conditions, with certain infections, and also with cancer (Figure 1B). Seipin is a conserved protein that plays an essential role in the formation and maintenance of LDs and also in the formation of adipose cells during development. Loss-of-function mutations in Seipin cause a severe form of lipodystrophy (Jin et al., 2020). At a cellular level, loss of Seipin leads to failure of LD maturation, most likely due to destabilized ER-LD contacts (Zoni et al., 2021). Seipin knockout cells have clusters of tiny nascent LDs and a few super-sized LDs, this bimodal distribution being very different from LDs around a normally distributed mean size seen in normal cells. LDs in Seipin knockout cells also show significantly greater intracellular motion compared to normal cells (Salo et al., 2016; Wang et al., 2016), but the significance of this motion remains to be determined.

Post-translational modifications (e.g., acetylation) of tubulin have been studied in the context of liver disease. Acetylation increases in cultured hepatocytes after ethanol exposure (Groebner and Tuma, 2015) and starvation (Geeraert et al., 2010). Alcohol induced acetylation of microtubules causes accumulation of large immobile LDs in hepatic WIF-B cells (Groebner et al., 2019). The activity of tubulin acetyltransferase αTAT-1 which is responsible for microtubule acetylation is regulated by AMPK-mediated phosphorylation (Mackeh et al., 2014). This is particularly intriguing since AMPK is considered a master regulator of lipid homeostasis, and alcohol-induced AMPK inactivation has been linked to hepatic steatosis (Donohue, 2007; You and Rogers, 2009). AMPK activation also causes an increase in detyrosination and reorganization of the microtubule cytoskeleton, as discussed above (Herms et al., 2015). Alcohol-induced microtubule acetylation may therefore lead to altered LD dynamics and alcoholic fatty liver disease. Unchecked lipid metabolism is a prominent marker for cancers. Cancer cells alter lipid metabolism to harness energy, membrane components and signalling lipids needed for their increased proliferation and invasion (Bian et al., 2021). Increased LD accumulation has been reported in various cancer cell types (Koizume and Miyagi, 2016). It is possible that LD motility is modulated in cancer, and this increases lipid exchange between LDs and other organelles to meet the enhanced energy demand. Indeed, LD velocity in cancer cells correlates with the severity of cancer (Nardi et al., 2019). The mechanism of this modulation of LD motion and its molecular basis is unknown.

### Lipid droplet dynamics during pathogen infection

Several intracellular pathogens target LDs via different mechanisms during their life cycle in the host cell. (Herker and Ott, 2012). The net result is rearrangement of LDs and/or pathogen-containing phagosomes to facilitate contacts between LDs and the pathogen. For instance, during *M. tuberculosis* infection, bacteria containing phagosomes migrate towards LDs for the engulfment of lipids (Peyron et al., 2008), whereas *Chlamydia* infection leads to redistribution of cellular LDs towards pathogen containing vacuoles (Wesolowski et al., 2017). Similarly in *M. leprae* infected Schwann cells, LDs relocate to intracellular bacterial inclusions induced by cytoskeletal modifications and PI3K signalling (Mattos et al., 2011). Electron microscopy in *Dictyostelium* amoeba infected with *M. marinum* shows a tight colocalization of LDs with pathogen-containing phagosomes, suggesting a mechanism for LD-phagosome fusion that enables transfer of LD contents into the phagosomal lumen (Barisch et al., 2015). Lipids are required by pathogens as a nutrient source, for vacuolar membrane dynamics, and for growth and replication (Fozo and Rucks, 2016). Hence, hijacking and translocation of LDs towards the pathogen may promote pathogen growth and survival (Figure 1B). A recent study however challenged the notion that LDs are always exploited by pathogens, demonstrating that LDs can also help in the immune response of the host. LDs in human macrophages harbour antimicrobial proteins on their surface which get upregulated when the host cell senses an infection. Upon challenge with lipopolysaccharide or *E. coli*, immune-active proteins get clustered around LDs. These proteins also uncouple LD-mitochondria interaction to reduce β-oxidation and increase LD-bacteria contacts (Bosch et al., 2020), for example, see Figure 1B. Notably, the anti-viral protein viperin that is induced in host macrophages upon hepatitis infection is also transferred to LDs from the ER after infection (Hinson and Cresswell, 2009).

Mechanisms by which infected cells accumulate antimicrobial proteins and mediate change in LD localization and inter-organelle contact formation are unclear. As mentioned above, a change in LD distribution can be attributed to differential recruitment of opposing (plus versus minus directed) motors on LDs, or to gross microtubule alterations, depending on the host and the invading pathogen. LD motion in Zika virus-infected primary astrocytes shows increased speed and displacement (Monson et al., 2021). Although these authors do not comment on which motors drive motion, increased LD motility facilitates inter-organellar communication for transfer of lipids and proteins required to mount an immune response against the virus. Another example where motors on LDs promote pathogen transport is during hepatitis-C virus (HCV) infection (Figure 1B). The HCV core proteins are recruited on LDs, causing dynein dependent LD transport towards the perinuclear region of infected cells (Boulant et al., 2008). In the next stage of its life-cycle, HCV utilizes the lipoprotein pathway to be secreted out as lipo-viral particles that infect other hepatocytes. During this stage, inhibition of kinesin-1 arrests the replication and secretion of HCV in the infected cell, possibly by disrupting the transport of viral proteins and other proteins responsible for lipoprotein lipidation (Rai et al., 2017). Motors also function in transport of pathogen-LD structures toward other organelles. Quantitative proteomics of LDs from live *M. tuberculosis* infected cells shows an accumulation of ADP-ribosylation factor-like protein 8B Arl8B (Menon et al., 2019), as compared to heat-killed bacteria or uninfected cells. Arl8B, along with SKIP protein can recruit the kinesin motor, mediating LD-lysosome interaction (Rosa-Ferreira and Munro, 2011).

### Cytoskeletal reorganization and its effect on LD motion in pathogen infection

Infection often leads to changes in microtubule dynamics, with several pathogens increasing the stabilization of microtubules. *Ectromelia* virus causes several cytoskeletal changes in host cells to facilitate transport and spread of the virus. These changes include loose and less intertwined microtubules, disruption of the microtubule organizing center (MTOC) and acetylation of microtubules to impart stability (Szulc-Dąbrowska et al., 2019). Similarly, HIV-1 infection also stabilizes microtubules by recruitment of CLIP-190 and targeting of mTOR (Naghavi, 2021). Even non-viral pathogens such as *Chlamydia* hijack Arf GTPases to cause post-translation modifications (mostly acetylation and detyrosination) of microtubules around the bacterial inclusion (Wesolowski et al., 2017). These modified microtubules can lead to differential localization, not only of LDs but also other organelles which may be required by the pathogen for its infectious cycle. For instance, upon *Chlamydia* infection, modified microtubules form a cage-like structure that helps in repositioning of Golgi around the pathogen inclusion body, which is required for imparting infectivity to the pathogen (Wesolowski et al., 2017). It will be interesting to examine whether LD-motion switches from dynamic microtubules to modified stable microtubules, which regulates LD interaction with a different set of organelles upon infection as compared to a healthy cell. Some of these cytoskeletal changes that cause LD accumulation, as in case of *Chlamydia* (Wesolowski et al., 2017), help in infection by the pathogen. It is possible that there is a competition between the host and the pathogen to alter the microtubule network, alter membrane contacts and recruit specific proteins on LDs that can reprogram LD function for increased survival. While different pathogens have different mechanisms to hijack the host machinery, LD function might be regulated differently in specific infections (Figure 1B).

### LDs as drug reservoirs–An opportunity for motor biologists?

**FIGURE 2:**
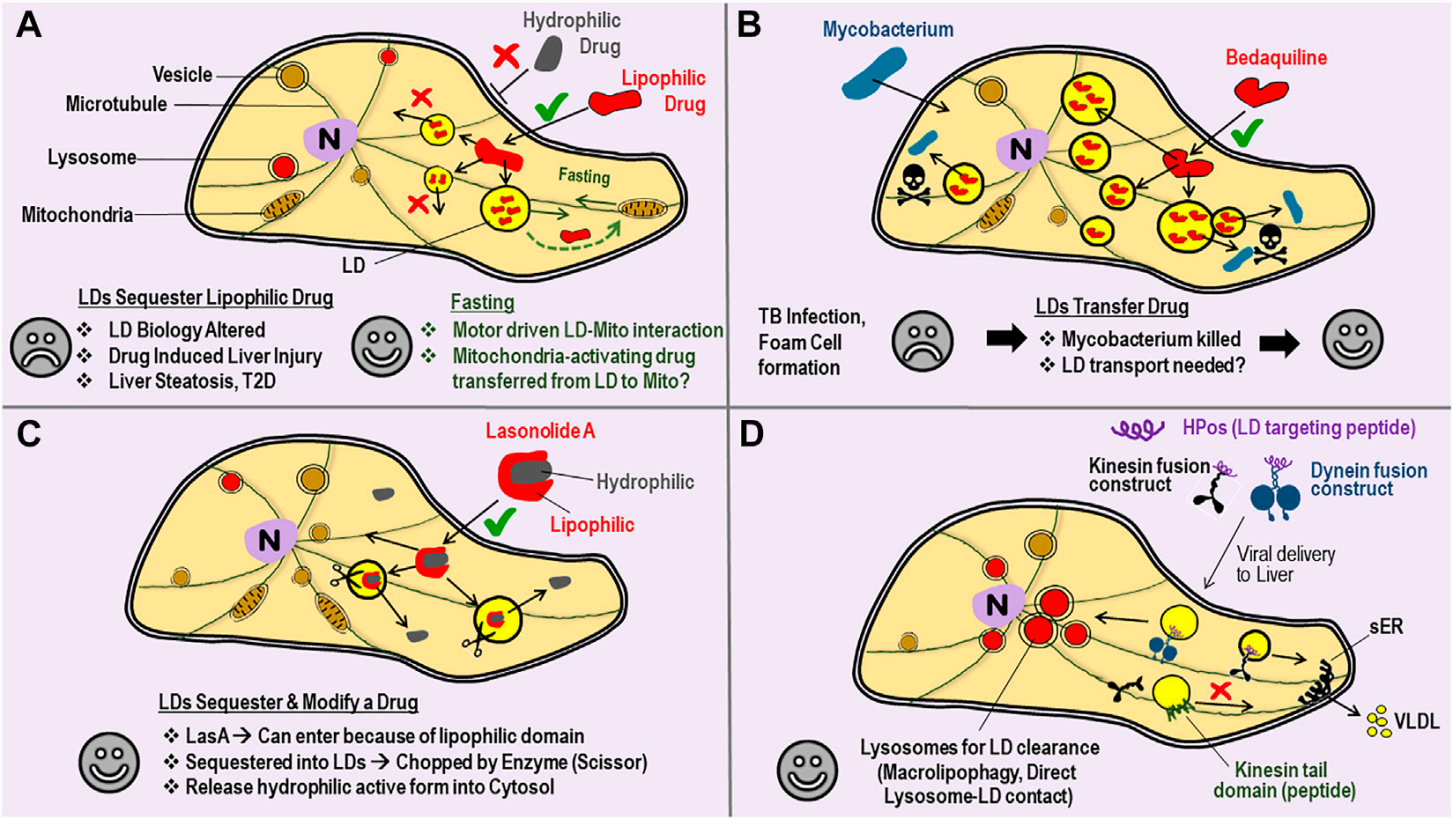
Lipid droplets can Sequester, Transfer or Modify Drugs–perhaps one could achieve targeted Drug Delivery by manipulating Motors on LDs. **(A)** A cartoon view of a Cell. Bilayer lipid membranes are shown with double-lines (e.g., plasma membrane, vesicle membrane) to distinguish from the monolayer lipid membrane on Lipid droplets (LDs). Entry into cells through the plasma membrane is often easier for lipophilic drugs (√ sign) as compared to hydrophilic drugs (× sign). Once inside, the lipophilic drug is sequestered away into LDs from where it cannot escape into cytosol, and therefore becomes ineffective as a drug. Such drug sequestration can lead to some of the undesirable consequences listed (sad face). Fasting/Starvation is known to increase LD-mitochondria interaction and consumption of LD-contents by mitochondrial β-oxidation. Perhaps hydrophobic drugs that can reverse mitochondrial degeneration can be packaged into LDs, and then delivered to mitochondria with a desirable outcome (happy face). **(B)** *Mycobacterium tuberculosis* infection causes LD accumulation (foam cell formation) in macrophages, with lipid-rich granulomas serving as a nutrient source for the bacteria. Bedaqulinine, a hydrophobic anti-tubercular drug, is sequestered away into LDs. The LDs act as a transferable reservoir of bedaquiline to kill *Mycobacterium* (skull sign). This function of LDs is in contrast to the sequestration of drugs by LDs described in panel-A. Perhaps LD-bacteria interactions (and therefore bedaquiline delivery) can be enhanced by targeted manipulation of motor proteins on the monolayer LD membrane. **(C)** Lasonolide-A (LasA) has both lipophilic and hydrophobic domains. LasA therefore enters through the cell membrane and accumulates into LDs, but there an enzyme (scissor) removes the hydrophobic part. Cleaved LasA is released into cytosol as a potent anti-cancer agent. This opens the possibility of designer drugs paired with cognate enzymes on LDs that can possibly be much more effective. **(D)** Targeted removal of kinesin-1 from LDs by using a kinesin tail domain peptide has been demonstrated (see main text). This intervention specifically blocks the transport of LDs to peripheral regions of hepatocytes inside the liver, so that the LDs can no more interact with the smooth-ER (sER). This treatment reduces serum triglycerides in the animal by reducing the triglyceride content of VLDL particles secreted from the liver. LD-targeting peptides (e.g., HPos) are known. HPos fusion constructs with motors can be targeted to the liver by adenoviral delivery, so that the motors accumulate specifically to LDs inside liver cells. A kinesin fusion construct could deliver LDs to the peripheral sER for increased clearance of LDs (*via* VLDL secretion) to ameliorate fatty liver conditions. A dynein construct could deliver LDs to lysosomes for LD-clearance via autophagic pathways.

Another interesting observation is the sequestration of drugs within LDs, usually with adverse, but sometimes desirable consequences. The lipophilic nature of some drugs enhances their uptake across the plasma membrane to increase the perceived bioavailability of such drugs (Figure 2A). But once inside the cell, the lipophilic drug may also get sequestered away into LDs to become unavailable for its intended purpose (Englinger et al., 2020). If motors could be activated on these LDs, one could possibly engineer targeted interaction of the LD with a pathogen, or with another relevant sub-cellular compartment, as the drug is being released. As an example, consider the known interaction between LDs and mitochondria driven by kinesin-2 in response to starvation (Herms et al., 2015). In this study the authors used siRNA against KAP3 to downregulate kinesin-2 function. Perhaps a hydrophobic drug that can reverse mitochondrial degeneration in Parkinson’s disease (Prasuhn et al., 2021) can be packaged into neuronal LDs, and then delivered efficiently to mitochondria when the patient is subjected to a fasting routine (Figure 2A). In another scenario a drug could get concentrated within LDs and possibly become more effective if such LDs are then made to interact, e.g., by regulating the motors on LDs, with another organelle such as a pathogen. The proliferation of *Mycobacterium* within LD-laden foamy macrophages of the host is well documented (Menon et al., 2019). Bedaquiline, a hydrophobic anti-tubercular antibiotic, is taken up by LDs inside host cells (Figure 2B). Interestingly, these LDs do not act as a sequestrator, but rather as a transferable reservoir that delivers bedaquiline to kill *Mycobacterium* in foamy macrophages (Greenwood et al., 2019). Another interesting example is Lasonolide A (LasA), a promising anti-cancer drug that is effective at low nanomolar concentrations. LasA accumulates into LDs after delivery, but there an enzyme cleaves and removes the hydrophobic part of the drug to now make LasA incompatible with LDs (Dubey et al., 2020). Cleaved LasA is released into cytosol as a potent anti-cancer agent (Figure 2C). These findings suggest the possibility of designer-drugs that can enter efficiently into cells because of their hydrophobicity, get sequestered away into LDs, but are then modified inside LDs for release into cytosol for a desired effect. The above discussed possibilities of drug delivery remain speculative at this stage, and would require the LD-specific regulation of motor protein activity, an aspect that appears possible for LDs as discussed next.

## Conclusion and outlook

Despite extensive research, tools to modulate the motion of a specific organelle in targeted manner are rare. However, this might become possible for LDs because protein attachment (and therefore motor-protein attachment) to the LD monolayer membrane could employ mechanisms different from bilayer membranes (Kory et al., 2016). Indeed, we demonstrated selective targeting of kinesin-1 on LDs. As shown in Figures 2A,D, a small peptide (the kinesin tail domain) was able to remove kinesin-1 from LDs in cultured hepatic cells (Kumar et al., 2019). This intervention caused a re-localization of LDs inside cells, but remarkably, had no effect on other bilayer vesicles that are also transported by kinesin-1 (e.g., lysosomes, mitochondria). How was such selectivity possible? Kinesin-1 can bind directly to phosphatidic acid (PA) on LDs (Kumar et al., 2019) and also to PA on bilayer vesicles (Wang et al., 2017). Perhaps kinesin-1 binding to the LD monolayer is weaker than bilayer membranes, causing removal of all LD-bound kinesins at a given peptide concentration, whereas some kinesins are still retained on the bilayer. Alternatively, kinesin-1 may have additional attachments to the bilayer membrane other than the kinesin tail domain. Regardless of the precise reason behind the LD-specific effect of kinesin tail domain peptide, the resultant re-localization of LDs correlated with reduced triglyceride secretion from cells, suggesting an intervention that can potentially lower serum triglycerides in blood. Mechanisms of protein recruitment to LDs are being revealed, and artificial peptides (e.g., HPos) that specifically target LDs have also been demonstrated (Kassan et al., 2013). If conjugated to such peptides, a motor could be targeted to LDs and then driven to cellular locations where specific LD-organelle interactions could be engineered (Figure 2D). In this context, an exciting study shows LD-lysosome interactions in hepatocytes wherein the LD contents are directly extruded into lysosomes for breakdown (Schulze et al., 2020). LDs and lysosomes are well known to localize to specific cellular locations in response to metabolic changes. Better understanding of motor protein function in these pathways, and targeted manipulation of the same could provide potential therapeutic benefits against liver steatosis, metabolic syndrome and infectious diseases.
